# Has FDA’s Drug Development Tools Qualification Program Improved Drug Development?

**DOI:** 10.1007/s43441-025-00790-2

**Published:** 2025-05-04

**Authors:** Felix Yang, Imein Bousnina, Anne Madej, Rasika Kalamegham

**Affiliations:** 1 Genentech, A Member of the Roche Group, South San Francisco, USA; 2 Howard University, Washington D.C., USA

**Keywords:** COA, Qualification, FDA, Primary/secondary endpoints, Context of use

## Abstract

**Background:**

The Drug Development Tools (DDTs) Qualification program creates a pathway to evaluate Clinical Outcome Assessments (COAs) that capture a specific concept of interest (COI) in a specified Context of Use (COU). If successfully qualified, a COA can be relied upon to measure a COI that has an application in drug development and regulatory decision-making. Thus, qualified COAs are important DDTs. This analysis aims to assess the Food and Drug Administration’s (FDA’s) performance reviewing applications in the COA Qualification Program, as well as the uptake of qualified COAs in drug development to date.

**Methods:**

In order to assess the use of qualified COAs in drug development, we analyzed the Summary Basis of Approvals (SBA) retrieved from Drugs@FDA and the COA compendium. The submission and review dates for the Letter of Intent (LOI), Qualification Plan (QP), and Full Qualification Package (FQP) steps were retrieved from Center for Drug Evaluation and Research (CDER) & Center for Biologics Evaluation and Research’s (CBER) database, as well as the FDA COA Qualification Program website.

**Results:**

Our analysis showed that 86 COAs were listed on the FDA COA website, with a majority of them being Patient Reported Outcomes (PRO). Completeness Assessment (CA) for each portion of submission, as well as review times for the LOI, QP, and FQP steps vary widely, with 46.7% of submissions having a review time exceeding the published targets. To date, 7 COAs (8.1%) have achieved qualification, and one (1.1%) has been denied after undergoing all steps for qualification. On average, it takes 6 years for a COA to be qualified. Our analysis of FDA’s approval documents shows that the Agency has relied on qualified COAs to support benefit-risk assessment of 11 medicines. Only three of the seven qualified COAs have been used to support benefit-risk assessment of medicines. The three qualified COAs that have been used are KCCQ, E-RS, and EXACT. Each of these has been used to support multiple indication claims. KCCQ – cardiomyopathy for 2 medicines, heart failure for 6 medicines; E-RS - chronic obstructive pulmonary disease (COPD) for 1 medicine; and EXACT – COPD for 3 medicines. Note: E-RS and EXACT were both used in aclidinium bromide/formoterol/fumarate. In each case they were used as secondary or exploratory endpoints, none as primary endpoints. Only 1 qualified COA was included in drug labels.

**Conclusion:**

The lengthy and unpredictable nature of the COA Qualification Program review timelines poses a risk for tool developers and sponsors intending to qualify a new COA, to use an existing COA or sponsors intending to qualify and use a new COA in the drug development process. Our findings show that, to date, the DDT Qualification Program has not significantly improved the inclusion of qualified COAs in clinical development plans to support regulatory decision-making and label claims, and therefore the impact of the pathway to facilitate the use of innovative tools has been limited. To improve the utility of this program, FDA should publicly share the timelines so participants can be better prepared to integrate into their development programs. Furthermore, FDA should clearly articulate how and when COAs can be used in drug development.

## Introduction

Modern-day drug development requires investments in methods [1, 2] that best capture and represent a drug’s effects on patients. For example, questionnaires for patients to report fatigue symptoms and daily physical limitations for heart failure [3]; report symptoms for cough, pain, dyspnea, fatigue, and appetite for non-small cell lung cancer (NSCLC) [4]; or reports of frequency, severity, and duration of COPD exacerbations [5].

For many diseases, there are currently no universally accepted methods to assess disease manifestation [1] and therefore drug efficacy. While there may be accepted approaches to measure clinical outcomes that indicate benefits for diseases that have a good understanding of the underlying biology, there is still a need to incorporate patient perspectives [6–8] on symptoms that matter most to their lives as well as their desired treatment outcomes. Further, for drugs that have novel mechanisms of action, there is a growing expectation to understand how patients perceive improvement/patient tradeoffs to provide a rationale for the endpoint development strategy. As a result, there is a growing emphasis on incorporating patients’ own perspectives in drug development [9] in order to evaluate a drug’s efficacy based on its improvement in patients’ daily life and functioning.

Recognizing the importance of incorporating the patient voice into drug development, the FDA initiated its Patient-Focused Drug Development (PFDD) [10] effort, as part of the Prescription Drug User Fee Act V (PDUFA V) [11], to capture patients’ voices and experiences in drug development. Compared to a biomarker, which is a defined characteristic that can be measured as an indicator of biological processes, a Clinical Outcome Assessment or COA, is a measure that describes or reflects how a patient feels, functions, or survives, capturing the full spectrum of a disease’s impact on a patient’s life [12]. The assessment can be made in various ways. If made through a report by a clinician, it is a Clinician Reported Outcome or ClinRO. If reported by a patient, whether by self-report or by interview, then it is a Patient Reported Outcome or PRO. If made by a non-clinician observer, it is Observer Reported Outcome or ObsRO. If it is a measurement based on standardized task(s) actively undertaken by a patient according to a set of instructions, it is a Performance-Based Assessment or a PerfO. PerfOs that leverage a Digital Health Technology (DHT) as a data-capturing tool are referred to by the FDA as DHT-Passive Monitoring COAs. The unifying factor between all the different types of COAs is that, after undergoing rigorous testing and validation, they are an accepted measure of how patients feel, function and/or survive and can be used in regulatory-decision making.

FDA’s Drug Development Tools (DDTs) Qualification Program was formalized via the passage of the 21st Century Cures Act (Cures Act) Sect. 3011 [13] in 2016. This program was established to allow the FDA to review and qualify DDTs such as biomarkers, COAs, and animal models [14]. We focused our analysis on COAs because of the growing importance of incorporating patients’ voices into drug development. Qualified DDTs are made publicly available to improve understanding of emerging regulatory science and to enhance drug development. Further, DDTs can be used in the review of regulatory applications such as Investigational New Drug (IND) Application, New Drug Application (NDA), or Biologics License Applications (BLA) to aid in assessment of a product’s benefit versus risk to patient.

**Fig. 1:**
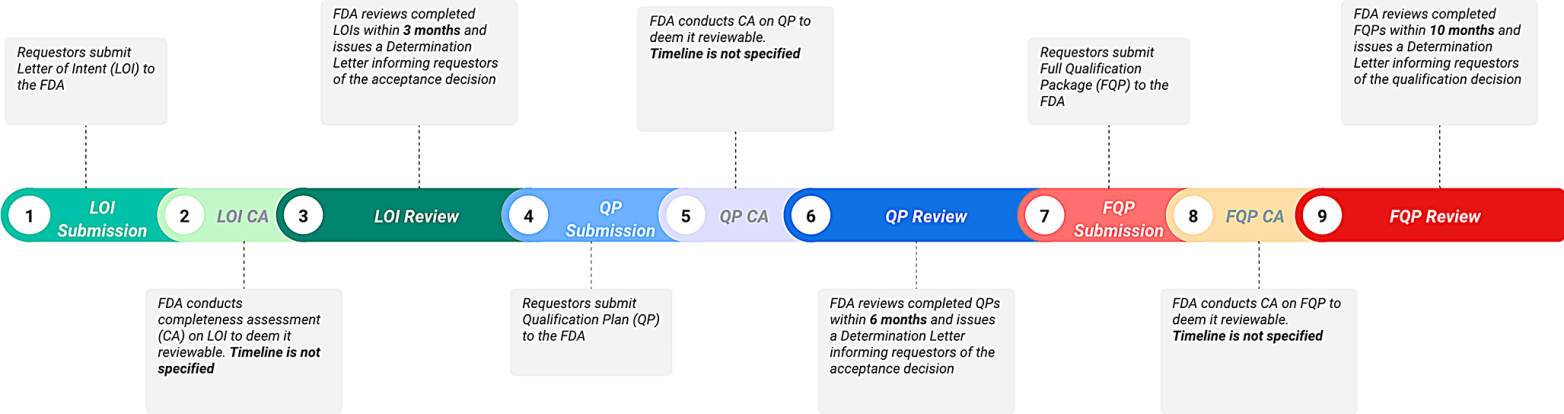
Process Map for DDT submission and qualification This process map is based on the FDA DDT Guidance [15] and provides the COA developer/requestor the steps they need to follow if they want to submit a DDT for review. Acronyms Used: LOI = Letter on Intent; CA = completeness assessment; QP = Qualification Plan; FQP = Full Qualification Package

As part of its efforts in codifying the DDT program, FDA issued a guidance document entitled “Qualification Process for Drug Development Tools” (the DDT guidance) in 2020 [15]. The DDT guidance outlines the step-by-step process, materials, and timelines for review (Fig. 1). In brief, the process involves; (1) Letter of Intent (LOI), (2) Qualification Plan (QP), and (3) Full Qualification Package (FQP). Prior to the review of each submission type, FDA will assess the completeness of the submission and readiness for review, which we are defining here as the Completeness Assessment (CA) step. Figure 1 shows the review timelines for LOI, QP, and FQP which are 3 months, 6 months, and 10 months, respectively. CA review timelines for each step are not specified in the DDT guidance. In the current process, there are no limits to the number of submissions per requestor, no submission fees, and no fixed deadlines by which a requestor needs to submit documents for subsequent steps after receiving the FDA determination letter.

According to FDA, COA qualification is a regulatory conclusion that the COA is a well-defined and reliable assessment of a specified concept of interest for use in adequate and well-controlled (A&WC) studies in a specified context of use (COU) [14]. COA qualification represents a conclusion that within the stated COU, results of assessment can be relied upon to measure a specific concept and have a “specific interpretation and application in drug development and regulatory decision-making.” Once a COA is qualified, it will be publicly shared via the FDA website and can subsequently be used not just by the requestor but by all sponsors and developers to support drug and biologic development [13, 14]. However, we note that, in determination letters from FDA, COAs are qualified as a measure for *exploratory use*.

Since the DDT program is now well-established, we decided to analyze its impact on drug development. Specifically, we chose to examine the performance and impact of the COA Qualification Program on innovative drug development, including the timeline and utilization of qualified COAs in drug clinical trials.

## Methods

In this analysis, we focused on COAs submitted between December 13, 2016, to October 10, 2024. To note, in our analysis, there are 22 COAs that were submitted prior to December 13, 2016, with no review dates available. We selected the start date based on the day that 21st Century Cures Act was signed into law, officially establishing the DDT qualification program. As the qualification program is ongoing, we selected an end date that would allow sufficient data to undertake our analysis. All our data were collected from publicly posted information via the FDA COA Qualification Program website [14].

We collected detailed information for COA submissions, including:

- COA submission number,
- disease/condition,
- concept of interest,
- COU,
- COA type, and.
- COA status.

COAs submitted prior to the formalization of the DDT Qualification Programs i.e. before December 13, 2016, received “507 Summary Response Letters”.

For each COA, submission and review dates for LOI, QP, and FQP were retrieved from the “Center for Drug Evaluation and Research (CDER) & Center for Biologics Evaluation and Research (CBER)’s DDT Qualification Project Search Database” [16]. Each submission type (LOI, QP, FQP) entails three different dates:

1. The date the COA developer submitted the document to FDA, i.e. what we are defining as the beginning of the CA phase (retrieved from the database’s date column for each document submission),
2. The date the document was deemed “reviewable” by FDA, i.e. what we are defining as the end of the CA phase (retrieved from each determination letter as the date FDA received the document), and.
3. The date FDA issues its decision on the status of the submission at a specific step of the process, i.e. accepted or denied for the LOI and QP steps, and qualified or not qualified for the FQP step.

We used embedded programs within Google Sheets for all calculations. The time taken by FDA for the CA was calculated by subtracting date 2) from date 1). The time taken by FDA for the review was calculated by subtracting date 3) from 2). Yearfrac function with a basis 1 was used to estimate the number of months it takes for review. Standard deviation (SD) was calculated using the STDEV.P function to quantify the variability in timeline and review performance.

For the qualified COAs, Summary Basis of Approvals and Prescribing Information retrieved from Drugs@FDA [17] and the COA compendium [18] were used to analyze their uses in clinical trials and product approval decisions. We used a commercially available Regulatory database (Cedience), to keyword search the qualified COAs in publicly available FDA review documents. The database contained all drug approval information from Drugs@FDA and CBER approved products in the time period of our analysis.

## Results

### Qualification Review Timelines

**Fig. 2:**
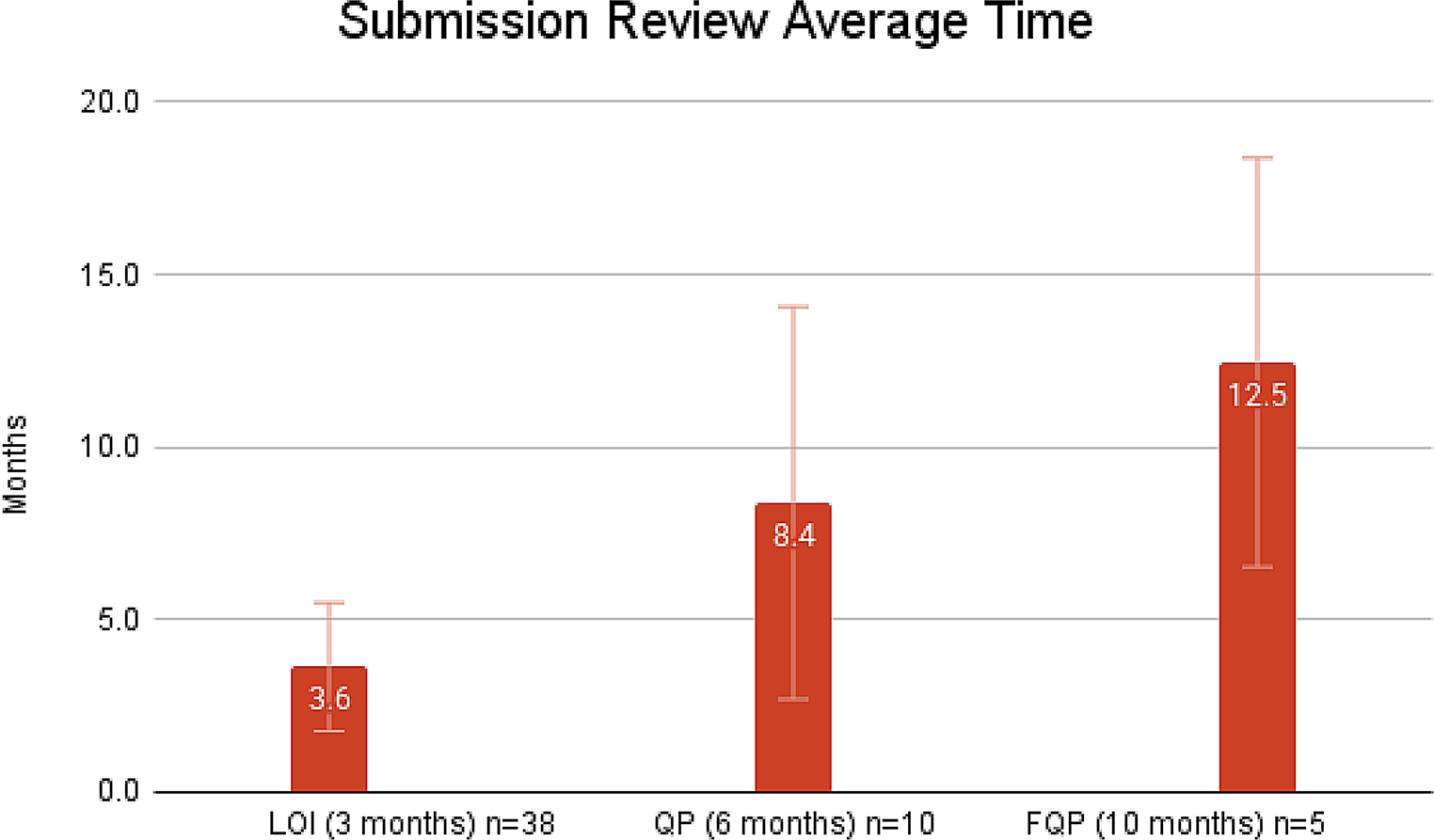
Average review time for each type of submission (LOI, QP, and FQP). For each submission, the average times are greater than the time FDA outlined in its DDT guidance. Error bar indicates SD

**Fig. 3:**
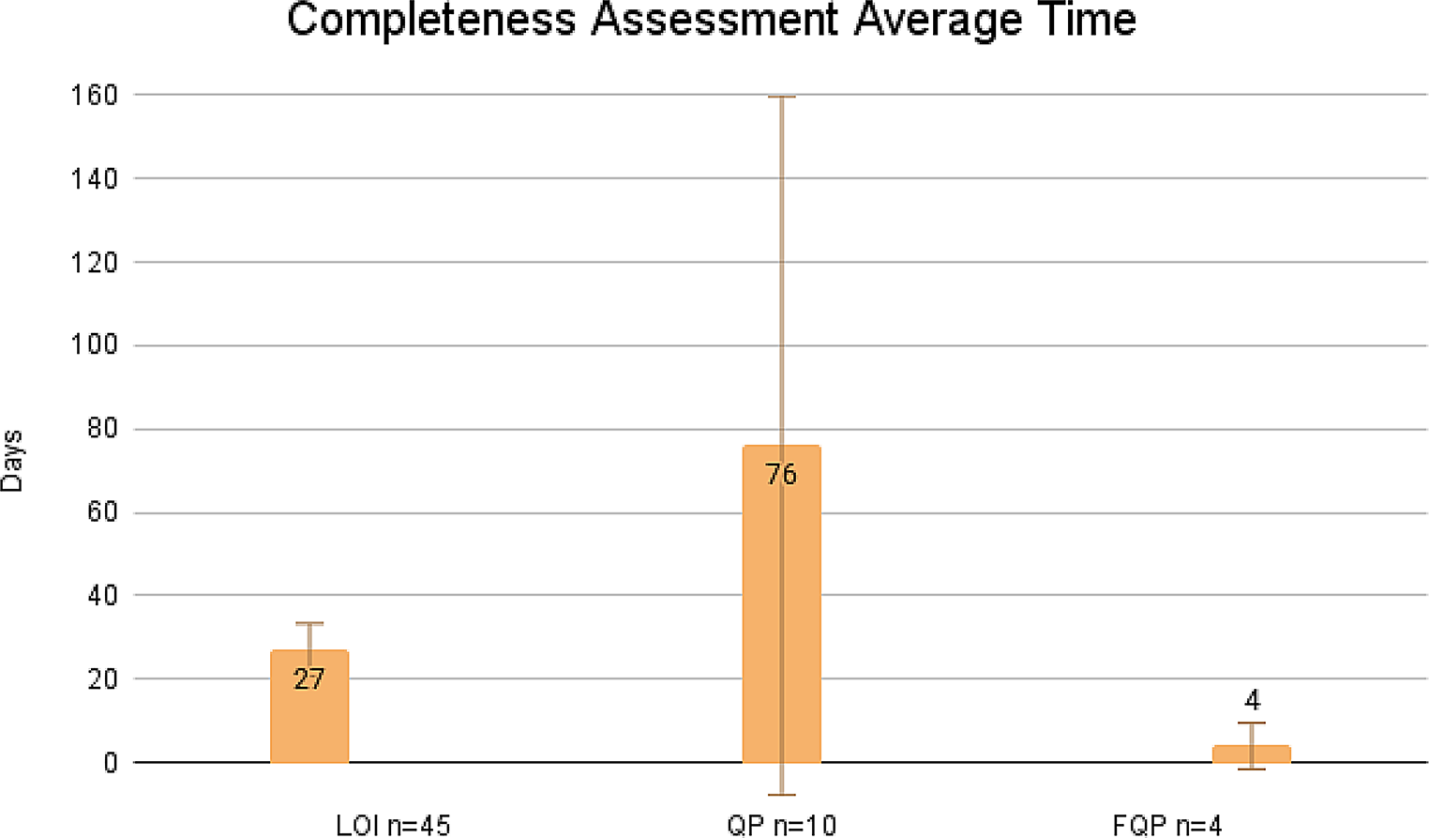
Average time for CA for each type of submission (LOI, QP, and FQP). CA timeline is not specified in FDA DDT Guidance. Error bar indicates SD

**Fig. 4:**
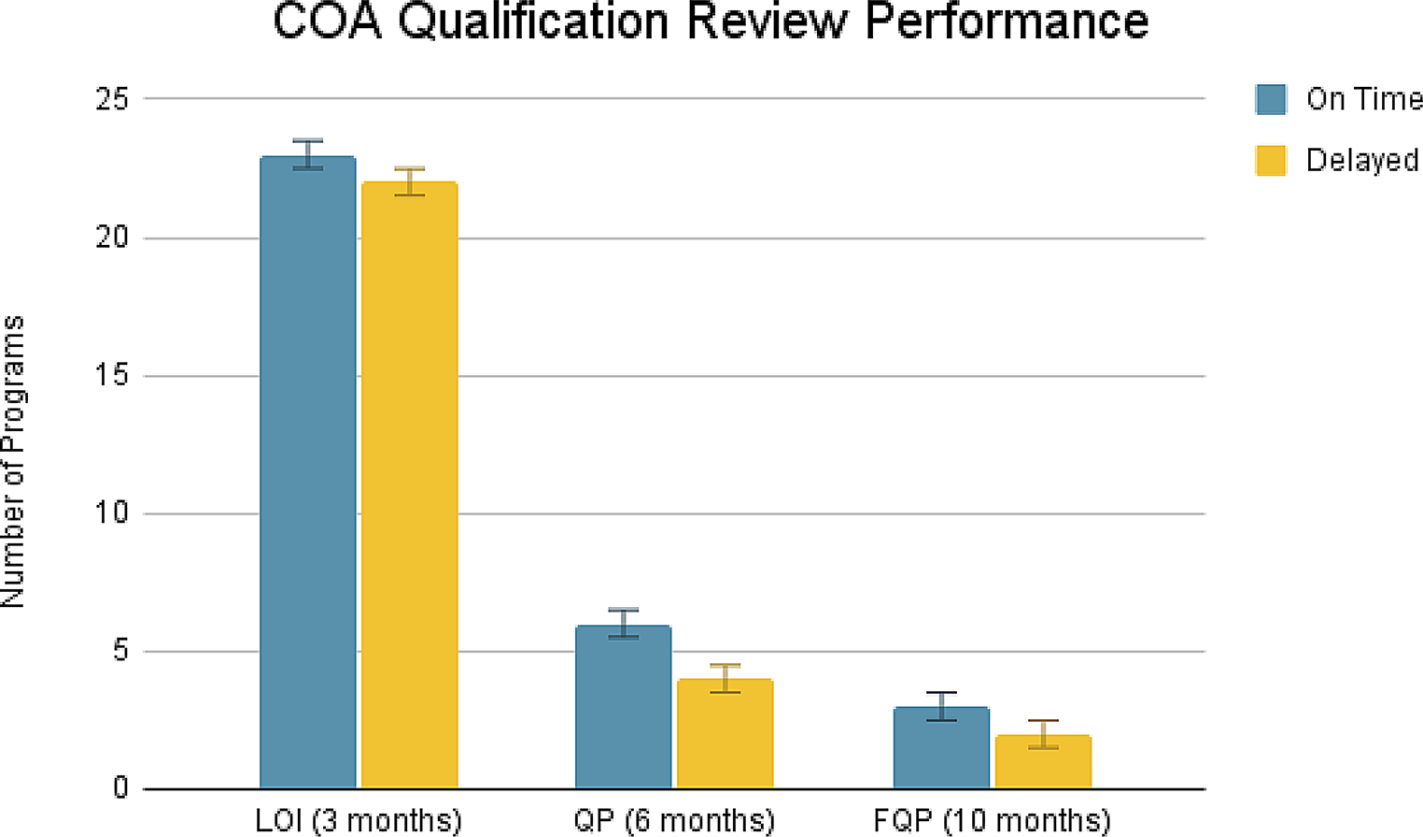
Number of submissions that were reviewed on time versus number of submissions that were delayed when compared against the timeline outlined in the DDT guidance. Error bar indicates SD

As of October 10, 2024, 86 COAs were published on the FDA COA website and DDT Qualification Project Search Databases. Of the 86 COAs, 60.5% are PROs (*n* = 52), 11.6% are PerfOs (*n* = 10), 9.3% are DHT-Passive Monitoring COAs (*n* = 8), 8.1% are ClinROs (*n* = 7), 3.5% are ObsROs (*n* = 3), and 7.0% are Mixed (*n* = 6; a combination of PRO, PerfO, DHT Passive Monitoring, ClinRO, and ObsRO). Of the 86 COAs, 9.3% (*n* = 8) had completed the final stage, i.e. FQP submission. 8.1% (*n* = 7) were qualified and 1 was denied after the final stage of review. To note, DDTs can be rejected at any stage of review. Our analysis showed that the average review timeline for each submission stage is as follows; 3.6 months for LOI, 8.4 months for QP, and 12.5 months for FQP. This is in contrast to the expected timelines as outlined in the DDT Guidance, e.g., expected timeline for LOI is 3 months; QP is 6 months; and FQP is 10 months (Fig. 2). Additionally, the time for review of each stage of the qualification program varies widely: the maximum time it took to review submissions for LOI, QP, and FQP were 11.43 months, 23.15 months, and 23.9 months, respectively. While the minimum time it took to review submissions for LOI, QP, and FQP were 1.73 months, 2.00 months, and 7.53 months, respectively. While not specified in the DDT Guidance, the average CA review time is 27 days for LOI, 76 days for QP, and 4 days for FQP (Fig. 3). The average time for qualification of a COA is 6 years. When compared against the timeline stated in the DDT Guidance, 51% of LOI, 60% of QP, and 60% of FQP, were reviewed on time (Fig. 4). Public-private partnership groups submitted the most COAs (43.0%, *n* = 37), followed by academia (41.9%, *n* = 36), and the private sector (15.1%, *n* = 13). Critical Path Institute has submitted the most COAs as a single requestor (22.1%, *n* = 19).

**Table 1:** Average review time for each type of submission (LOI, QP, and FQP). Standard deviation (SD), mean absolute deviation (MAD), and variance were calculated

Average Review	Months	SD	MAD	Variance
LOI (3 months) *n* = 38	3.6	1.86	1.26	4
QP (6 months) *n* = 10	8.4	5.69	4.32	36
FQP (10 months) *n* = 5	12.5	5.92	4.58	44

## Analysis of Qualified COAs

Seven COAs (8.1%) have been qualified as of October 10, 2024 (Table 1). According to their Qualification Determination Letters, all 7 COAs were qualified as a measure. The exact language used in the determination letters is: “*qualified for exploratory use […] to measure […] in clinical studies. Additional development work is needed to further assess measurement properties*” [19]. This means COAs are qualified as measures to be used as exploratory endpoints until further data is collected to prove their reliability as future key endpoints. We also note FDA does not define “measure” in the determination letter and how a qualified COA will translate to a primary, secondary, or exploratory endpoint, contributing to regulatory decision making. Thus, none of the qualified COAs were described in their determination letters as qualified for use as a key primary or secondary endpoint (Tables 2, 3 and 4).

Three qualified COAs were used in the drug clinical development plans to support benefit-risk assessments as secondary and exploratory endpoints, but none were used as primary endpoints. Amongst them, only 1 (KCCQ) was included in several drug labels supporting benefit claims. None of the qualified COAs determination letters have been updated to reflect the impact further uses in drug development have had on their qualification status as permitted by the DDT guidance. Per the DDT Guidance, original requestors are able to submit a QP to modify the qualified DDT without changes to the COU, e.g., longitudinal data for COA. A third party, who is not the original requestor, may submit a LOI to the FDA along with supporting data and rationale for additional qualification. However, it is unclear what the process is to modify the qualified DDT to reflect its acceptable use as a key primary or secondary endpoint as the DDT Guidance does not address it.

**Table 2:** Average time for CA for each type of submission (LOI, QP, and FQP). Standard deviation (SD), mean absolute deviation (MAD), and variance were calculated

Average CA	Days	SD	MAD	Variance
LOI *n* = 45	27	23.37	20.68	558.59
QP *n* = 10	76	83.84	62.98	7809.57
FQP *n* = 4	4	5.5	4.75	40.33

**Table 3:** Number of submissions that were reviewed on time versus number of submissions that were delayed when compared against the timeline outlined in the DDT guidance. Standard deviation (SD), mean absolute deviation (MAD), and variance were calculated

Review Performance	On Time	Delayed	SD	MAD	Variance
LOI (3 months)	23	22	0.5	0.5	0.25
QP (6 months)	6	4	0.49	0.48	0.24
FQP (10 months)	3	2	0.49	0.48	0.24

**Table 4:** Seven qualified COAs as of October 10, 2024

COA Name	COA Requestors as cited in Determination Letters	COA Type	Context Of Use	Qualification Date	Qualification Process Duration	Accepted as Primary Endpoint In Clinical Study (Y/*N*)	Drugs for which COA was a Secondary Endpoint Found in Label	Drugs for which COA was a Secondary or Exploratory Endpoint not Found in Label
DIBSS-C (000005) [20]	Critical Path Institute	PRO	Patients 18 years and older with a diagnosis of irritable bowel syndrome with constipation	12/18/2020	5.60 years	N	N/A	N/A
KCCQ (000084) [3]	John Spertus	PRO	Patients with congestive heart failure	4/9/2020	4.30 years	N	tafamidis meglumine (cardiomyopathy), mavacamten (cardiomyopathy), sotagliflozin (heart failure)	Ivabradine (heart failure), dapagliflozin (heart failure), Sacubitril/valsartan (heart failure), vericiguat (heart failure), empagliflozin (heart failure)
ANSD (000006) [21]	Critical Path Institute	PRO	Adolescent & adult patients with asthma	3/28/2019	8.36 years	N	N/A	N/A
NSCLC-SAQ (000009) [4]	Critical Path Institute	PRO	Patients 18 years and older with Stage IIIB or IV non-small cell lung cancer	4/4/2018	6.69 years	N	N/A	N/A
SMDDS (000008) [22]	Critical Path Institute	PRO	Adults with major depressive disorder	11/27/2017	7.19 years	N	N/A	N/A
E-RS: COPD (000017) [19]	Evidera	PRO	Adult outpatients with stable chronic obstructive pulmonary disease	3/10/2016	5.02 years	N	N/A	Aclidinium bromide/formoterol fumarate (COPD)
EXACT (000003) [5]	Evidera	PRO	Outpatients with acute bacterial exacerbations of chronic bronchitis.	1/9/2014	Unclear	N	N/A	Glycopyrrolate (COPD), aclidinium bromide/formoterol fumarate (COPD), budesonide/glycopyrrolate/formoterol (COPD)

### Analysis of 2 Qualified COAs with Most Utilization by FDA

Our analysis showed that there were 2 COAs that were used extensively by FDA to make regulatory decisions- namely KCCQ and EXACT.

### KCCQ

**Fig. 5:**
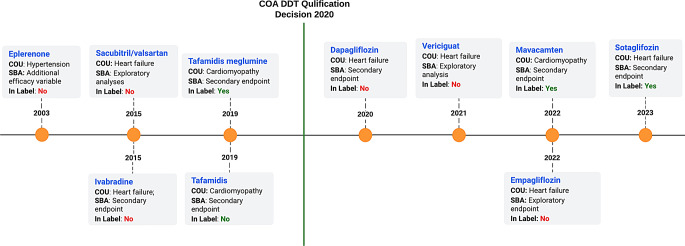
Application of KCCQ in different benefit-risk assessments of drugs over time as found in SBAs including COU and if it was included in the drug label

The Kansas City Cardiomyopathy Questionnaire (KCCQ) [3] is a PRO qualified for patients with congestive heart failure to measure “patients’ health status, including their symptoms (frequency and burden), physical and social limitations, and quality of life impact due to the heart failure syndrome.” It was submitted to the COA DDT Qualification Program on December 21, 2015, and qualified on April 9, 2020, taking 4.30 years for qualification. KCCQ was originally published [23] in 2000 and had been used in benefit risk assessment for new drugs prior to receiving its qualification status. KCCQ was used as an additional efficacy variable for eplerenone (2003), exploratory analyses for sacubitril/valsartan (2015), and secondary endpoint for ivabradine (2015), tafamidis meglumine (2019), tafamidis (2019), and dapagliflozin (2020). Despite the use of KCCQ as a secondary endpoint in benefit risk assessment of multiple new drug applications, KCCQ was not qualified as such. It was qualified as a “measure” with additional development work needed to further assess measurement properties in drug development programs. Post qualification, KCCQ was used as exploratory analyses for vericiguat (2021) and empagliflozin (2022), and as a secondary endpoint for mavacamten (2022) and sotagliflozin (2023) (Fig. 5). Despite these numerous uses in drug development, KCCQ’s qualification status has not been updated to prove its potential increased reliability for regulatory decision-making. Of note, KCCQ is the only COA qualified to date that has been included in the drug label of multiple drugs (tafamidis meglumine, mavacamten, and sotagliflozin).

### EXACT

The EXAcerbations of Chronic Pulmonary Disease Tool (EXACT) [5] is a PRO qualified for symptoms of acute bacterial exacerbations of chronic bronchitis in patients with chronic obstructive pulmonary disease (COPD). While the LOI submission date was unclear based on FDA’s database, it was qualified on January 9, 2014. EXACT was used as an exploratory endpoint for glycopyrrolate (2017) and aclidinium bromide/formoterol fumarate (2019), and as a secondary endpoint for budesonide/glycopyrrolate/formoterol (2020). Based on the SBA for aclidinium bromide/formoterol fumarate [24], the sponsor inquired about using EXACT as a secondary endpoint, but FDA stated that it was more appropriate as an exploratory endpoint at the time. Although it had been 5 years since its qualification and there had already been one use of EXACT as an exploratory endpoint in a drug development program (glycopyrrolate), EXACT was not accepted to be used as a secondary endpoint for aclidinium bromide/formoterol fumarate. In spite of the availability of EXACT as a qualified PRO, a non-qualified PRO, St. George’s Respiratory Questionnaire (SGRQ), was included in the drug label [25–27] of all three drugs. SGRQ was considered a secondary endpoint and therefore included in the labels of the drugs.

## Discussion

We examined the qualification status of 86 COAs that were submitted to the FDA’s DDT qualification program as of October 10, 2024. Of the 86, 7 were qualified. Of note, all 7 qualified COAs were submitted to FDA prior to the passage of the Cures Act in 2016. None of the COAs submitted post-passage of the Cures Act have been qualified, as of October 10, 2024. One COA submitted on November 3, 2015, was denied at the final stage of review. This was particularly surprising because the DDT qualification program is designed to allow requestors to interact with FDA throughout the process such that advice and feedback can be given to ensure a successful outcome. It is not clear why the COA was rejected after all the milestones were met. No new COAs have been qualified since 2020.

Our results show that the timeline for qualification of a COA can vary widely. Examination of each stage of the review process shows that the FDA does not always meet the timeline set forth in the DDT Guidance [14], with nearly half of the submissions being reviewed late. This is noteworthy in part because the timelines were decided upon and established by FDA. Thus, it is perplexing to see the Agency not meet these timelines that it set for itself. Lastly, we note that the review timeline of the CA step (step 2, 5, 8 in Fig. 1) varies widely across the 86 submissions currently in the program. The lengthy and unpredictable nature of the review timeline poses a risk for developers planning to develop, qualify and use newly qualified COAs in drug development programs.

When we examined the utility of the qualified COAs in the benefit-risk assessment of drugs, we found that although considered successfully qualified, the determination letters stated that each COA was qualified as “measures” that required additional development work to further assess their measurement properties. This means that sponsors would need significant additional evidence generation within a drug clinical development plan for the qualified COA to eventually be accepted by FDA for use as a key endpoint for benefit-risk assessment. We also note that FDA does not define “measures” within the DDT qualification program and therefore it is unclear if the term is understood and leveraged consistently across drug review divisions. Not surprisingly, we found that even when qualified, COAs have only been accepted as exploratory or secondary endpoints. None of the COAs qualified via the DDT pathway thus far have been accepted as a primary endpoint. Furthermore, none of the qualified COAs appear on the FDA’s published table of surrogate endpoints that can be the basis of drug approval or licensure. This is despite some having been used post-qualification in multiple clinical development plans as exploratory or non-key secondary endpoints.

To date, the great majority (85.7%) of qualified COAs are not found in drug labels. However, it is unclear whether this can be fully attributed to drug sponsors or the FDA. During the clinical development phase of a drug, the choice of clinical endpoints and their hierarchy in the clinical trial statistical plan is ultimately the sponsor’s decision, with the opportunity to seek feedback from the Agency on its acceptability for regulatory decision-making during the review. This feedback opportunity allows early input from the FDA to determine whether a qualified COA is an appropriate endpoint for it to rely upon for regulatory decision-making in the context of the drug’s review. In one example [24] found in the Drugs@FDA database dating from 2017, a sponsor inquired about the use of E-RS as a secondary endpoint in a phase 3 clinical study for patients with stable COPD. E-RS [19] had been qualified since 2016 for the following COU “exploratory use as a PRO instrument to measure respiratory symptoms of stable COPD in clinical studies.” During the interaction with the sponsor, FDA stated that E-RS was best used as an exploratory endpoint instead as “[PROs] are not reliable for studies of patient populations with moderate to severe disease”. Ultimately, the utility of COAs “qualified as measures” for drug development is unclear, especially given the uncertainty about FDA’s willingness to rely on qualified COAs as key primary or secondary endpoints for regulatory decision-making. Interestingly, we found multiple examples of drug labels [28, 29] that included COAs that were not qualified via the COA Qualification Program, highlighting that COAs can be deemed reliable for regulatory decision-making and benefit-risk assessment without being qualified. Thus, it appears that FDA is not averse to utilizing COAs for benefit/risk assessment per se. It does, however, raise questions about the utility of the qualification program since none of the COAs qualified via that pathway have been accepted as a key measure for regulatory decision-making.

Interestingly, FDA’s Center for Devices and Radiological Health (CDRH) hosts its version of a qualification program for medical device development tools (MDDT qualification program). Similar to the CDER/CBER DDT qualification program, the MDDT qualification program [30] is a way for FDA to qualify biomarkers, COAs, and animal models to be used for medical device development. For example, KCCQ, discussed above, underwent the MDDT qualification program in addition to the DDT qualification program. According to the FDA, a qualified tool under the MDDT program “will be accepted by the FDA without the need to reconfirm the suitability and utility of the tool within the same [COU]” for the regulatory review of medical devices. KCCQ was qualified for use as a primary or secondary endpoint in a feasibility or pivotal clinical trial as stated in the MDDT qualification decision summary [31]. This is in contrast to the language in the DDT qualification determination letters where COAs are qualified as a measure.

FDA has promoted the use of the DDT qualification program in many ways including workshops [32] and the release of a series of four guidance documents [33] to support the development process of a COA. Guidance 1 [34] focuses on the quantitative and qualitative methodologies on collecting patient experience data and ensuring that the data is representative of the intended population. Guidance 2 [35] discusses qualitative methods to identify the aspects of disease that are important to patients, such as disease symptoms or daily life functioning. Guidance 3 (draft) [36] provides recommendations on developing a fit-for-purpose COA in clinical trials with specific concept of interest (COI) and COU. Lastly, guidance 4 (draft) [37] addresses ways to incorporate COA as an endpoint in clinical trials for regulatory submissions. These guidances set FDA’s expectations for the development of a reliable COA to be used for regulatory decision making. They also demonstrate the agency’s belief that COAs can be an important and reliable tool to assess patient experience which in turn can help inform regulatory decision-making. However, to date, these guidances have not translated into increased uptake of COAs within the qualification program.

While the qualification of a novel COA is not required for it to be used as an endpoint for drug development, developing and validating a novel COA solely within the context of a drug program has many limitations for the community and even the COA developers themselves. COAs that were fully developed in the context of an IND have limited uses beyond that specific drug program, as the data used to develop and validate them is closely tied to the drug development’s data protected via the NDA/BLA. This greatly hinders the uptake of novel COAs across multiple sponsors. The intent of Congress when creating the DDT qualification program was to facilitate the use of qualified COAs by the entire scientific community. COAs, when qualified, become publicly available for all stakeholders to use and can be “relied upon” within the stated COU [14]. COAs are particularly important to support patient-focused drug development by measuring and describing how a patient feels, functions, or survives. Their broader use in drug development would allow patients to better interpret treatment benefits for shared decision-making with their healthcare providers.

Our findings show that the COA Qualification Program, to date, has not enabled the inclusion of COAs in drug labels and therefore has largely not supported the use of innovative tools in the benefit-risk assessment of drugs. Actionable recommendations for improvement include:

1. Publishing actual qualification timelines for COAs such that sponsors can factor it into their molecule development timelines;
2. Including a list of qualified COAs as surrogate endpoints that can be used in the benefit risk decision by FDA; and.
3. Encouraging sponsors and FDA to publish their experience and best practices with COAs and the qualification program.

## Conclusion

Our analysis reviews the performance and utility of the COA Qualification Program. The DDT Qualification Program was formally established by The United States Congress via passage of the 21st Century Cures Act, Sect. 3011. Congress recognized the value of enabling the translation of basic research advances into novel drug development tools to enhance and expedite the next generation of medical products to improve patient’s lives. Congress also acknowledged the need to leverage the experience and expertise within the FDA to enable the development of reliable tools for drug development by establishing this pathway [38].

The establishment of a formal program to develop novel tools raised expectations amongst all stakeholders; including academia, industry, patient advocates and others; that there would be better, more reliable tools to measure responses for interventions and that in turn, these drug development tools will enable faster development of medicines. Our analysis however shows the opposite. We find that the program itself is highly variable in its review timelines despite said timelines having been established by the FDA itself. The parallel to a new molecular entity (NME) is striking since there are established milestones as part of the DDT qualification program that somewhat mirror the development milestones for NMEs. In theory, these parallel tracks for development should enable a sponsor to assess whether or not to employ a COA in their clinical development plan. However, as our analysis has shown, timelines for the qualification of COAs are unreliable and therefore participating in the qualification program can be a perceived risk for tool and drug developers. Our analysis has also shown that qualified COAs have never been accepted as a primary endpoint in a study. Lastly, we show that despite being qualified, COAs’ inclusion in drug labels is extremely limited. Of significant note, some COAs that have not gone through the qualification pathway are found in drug labels. In the example of aclidinium bromide/formoterol fumarate in COPD, the sponsor inquired about the use of EXACT as secondary endpoint. FDA did not agree stating that “[it is] best used as exploratory endpoint at this time […] In general, patient reported symptom questionnaires are not reliable for studies of patient populations with moderate to severe disease”. The adoption of qualified COA may depend on several factors including the disease state and the severity of disease upon FDA discretion.

COAs are a powerful tool to measure how patients feel, function and survive. Measuring how patients live and navigate their daily life whilst shouldering the burden of disease is the essence of patient focused drug development. Many types of illnesses and diseases that clinicians treat do not lend themselves well to measurement through traditional clinical endpoints. Industry, FDA, and the clinical community are united in their desire to improve treatment options for patients. That means understanding and measuring benefits that cannot be measured by clinical endpoints alone and leveraging additional, scientifically robust tools such as COAs to assess benefit/ risk. Thus, the value of developing and qualifying COAs is the ability to deploy them in molecule development programs, especially for diseases that have few or no traditional clinical endpoints. In addition to helping regulatory decision-making, having information about COAs will allow patients and clinicians make better-informed treatment decisions at the bedside.

However, the unpredictable qualification review timeline poses a risk for planning and submission of new COAs to the program. This could represent a missed opportunity to encourage capturing data that reflects how a patient feels, functions, or survives. Getting the full spectrum of a disease’s impact on a patient’s life can help healthcare providers and patients make informed decisions about treatment options and desired outcomes. Sharing how a medicine might impact the daily functioning of a patient is information that can be vitally important when the patient, their caregivers, and loved ones make treatment decisions. The DDT Qualification Program has not significantly driven the inclusion of qualified COAs in clinical development plans to support regulatory decision-making and label claims, and therefore the impact of the pathway to facilitate use of innovative tools has been limited. Congress intended the program to provide clarity and predictability for stakeholders developing such DDTs, but FDA’s implementation of the program has fallen short of these goals thus far.

## Data Availability

All data are collected via the Food and Drug Administration (FDA) Clinical Outcome Assessment (COA) Qualification Program website (https://www.fda.gov/drugs/drug-development-tool-ddt-qualification-programs/clinical-outcome-assessment-coa-qualification-program) and CDER & CBER’s DDT Qualification Project Search database (https://force-dsc.my.site.com/ddt/s/).
